# Treatment Strategies for Hepatic Metastases From Pancreatic Cancer in Patients Previously Treated With Radical Resection Combined With Intraoperative Radiation Therapy

**DOI:** 10.1155/1994/61323

**Published:** 1994

**Authors:** Hiroshi Takamori, Takehisa Hiraoka, Keiichirou Kanemitsu, Tatsuya Tsuji, Naoyuki Saito, Hidefumi Nishida, Hisashi Sakaguchi, Yoshimasa Miyauchi

**Affiliations:** First Department of Surgery Kumamoto University School of Medicine Honjo, Kumamoto 1-1-1, 860 Japan

## Abstract

Since 1984, we have performed extended radical resection combined with extended intraoperative radiation therapy (IORT) for pancreatic cancer. This approach has provided a dramatic improvement in long-term survival and control of local recurrence. Hepatic metastases, however, remain an unsolved problem. Among patients with this combined therapy, we found hepatic metastases in 8 of 22 patients postoperatively. Four of these 8 were considered candidates for further therapy and underwent treatment for their hepatic metastases, the other 4 had too extensive disease. Two patients with multiple hepatic metastases underwent percutaneous ethanol injection therapy and chemotherapy, but they died within a year. Two patients with a solitary hepatic metastases underwent hepatic resection. One patient died two years and six months after the first operation because of multiple metastases in the liver and both lungs, while the other patient is still alive over six years after the first operation with an excellent performance status. When a patient has no local recurrence and a solitary metastasis in the liver, surgical resection of the liver metastasis should be performed.


*HPB Surgery*, 1994, Vol. 8, pp. 107–110
Reprints available directly from the publisher
Photocopying permitted by license only

© 1994 Harwood Academic Publishers GmbH
Printed in Malaysia

### Treatment Strategies for Hepatic Metastases from Pancreatic Cancer in Patients Previously Treated with Radical Resection Combined with Intraoperative Radiation Therapy

HIROSHI TAKAMORI, TAKEHISA HIRAOKA, KEIICHIROU KANEMITSU, TATSUYA TSUJI, NAOYUKI SAITO, HIDEFUMI NISHIDA, HISASHI SAKAGUCHI and YOSHIMASA MIYAUCHI

First Department of Surgery, Kumamoto University School of Medicine, Honjo 1-1-1, Kumamoto, 860 Japan

Since 1984, we have performed extended radical resection combined with extended intraoperative radiation therapy (IORT) for pancreatic cancer. This approach has provided a dramatic improvement in long-term survival and control of local recurrence. Hepatic metastases, however, remain an unsolved problem. Among patients with this combined therapy, we found hepatic metastases in 8 of 22 patients postoperatively. Four of these 8 were considered candidates for further therapy and underwent treatment for their hepatic metastases, the other 4 had too extensive disease. Two patients with multiple hepatic metastases underwent percutaneous ethanol injection therapy and chemotherapy, but they died within a year. Two patients with a solitary hepatic metastases underwent hepatic resection. One patient died two years and six months after the first operation because of multiple metastases in the liver and both lungs, while the other patient is still alive over six years after the first operation with an excellent performance status. When a patient has no local recurrence and a solitary metastasis in the liver, surgical resection of the liver metastasis should be performed.

KEY WORDS: Pancreatic cancer   hepatic metastases   extended radical resection   intraoperative radiation therapy

### INTRODUCTION

Carcinoma of the pancreas represents one of the most challenging diseases faced by abdominal surgeons. The long-term survival of patients following resection remains poor[1]. Even after radical resection, we very often find local recurrences and hepatic metastases at autopsy.

In order to control local recurrence after radical resection for pancreatic cancer, we have performed extended radical pancreatectomy combined with intraoperative radiation therapy (IORT) since 1984. The five year cumulative survival rate of these patients was 33.3 percent. Four autopsies were permitted among 14 patients. Three of four autopsied cases showed no evidence of local recurrences. This approach has provided a dramatic improvement in long-term survival and control of local recurrence compared with other therapies[2].

Even after control of local recurrence by this combined therapy, we have found hepatic metastases, these have often been multiple and the patients have had a poor prognosis. However one patient died with only two localized liver metastases seen at autopsy in 1986. Since that time we have taken surgical treatment for patients with localized metastases into careful consideration. We present a treatment strategy for such hepatic metastases.

*Corresponding author:* Takehisa Hiraoka, First Department of Surgery, Kumamoto University school of Medicine, Honjo 1-1-1, Kumamoto, 860 Japan, Telephone: (Japan) 96-344-2111, Fax: (Japan) 96-363-9674

### PATIENTS AND METHODS

During the period from 1984 to 1992, we performed extended radical resection combined with extended IORT on 22 patients. As described previously[2], this combined therapy involves the dissection of the juxtaregional and regional lymph nodes together with the connective tissue around the aorta extending from the diaphragm to the inferior mesenteric artery. Following dissection and/or vascular reconstruction, a dose of 30 Gy with 9 MeV electron beam radiation was administered to the operative field including the paraaortic area from the diaphragm to the inferior mesenteric artery using a special applicator that could be varied in size to accommodate the operative field on an individual basis. In this series, we found liver metastases in 8 patients after the operation. Four of these 8 patients underwent treatment for their liver metastases, the other 4 patients had multiple metastases in multiple organs (Table 1).

### CASE REPORTS

### Case 1

A 70-year-old man was diagnosed as having cancer of the neck of the pancreas. He underwent the combined therapy as described above. On histopathological examination, the tumor was a stage III moderately differentiated tubular adenocarcinoma (pT2, pN1, pM0). At the margin of the resected specimen, cancer cells were found histologically. This operation was classified as a nonradical operation. A computed tomography (CT) scan 22 months after the operation revealed a single 3 cm low density area in the segment VI of the liver. We performed percutaneous ethanol injection therapy for this lesion, but the metastatic lesion did not change over ten months, and no new lesions appeared in the liver. Since no local recurrences were detected, he underwent partial hepatectomy for resection of the metastasis. The histopathological diagnosis of this metastasis was a well differentiated tubular adenocarcinoma with invasion into Glisson's sheath. A CT scan 17 months after the hepatic resection revealed a new 3 cm low density area in the remnant segment VI of the liver, but no evidence of any local recurrences existed. Another partial hepatectomy was performed. At surgery, the tumor was noted to have invaded into the right kidney, ascending colon, and right diaphragm. We performed a partial resection of these organs. By histopathology, the tumor was a well differentiated tubular adenocarcinoma, indicating metastases from the original pancreatic cancer. He has recovered with an excellent performance status. He is still alive over six years after the first operation.

### Case 2

A 58-year-old man was diagnosed as having cancer of the head of the pancreas, and he underwent the combined therapy already described. By histopathology, the tumor was a stage III moderately differentiated tubular adenocarcinoma (pT2, pN1, pM0). His operation was nonradical histologically because cancer cells were observed at the resection margin of the specimen. A CT scan one year and nine months after the resection showed a low density area of 2 cm in diameter in the segment VI of the liver, and a coin lesion in the segment VI of the right lung. For the hepatic metastasis, he underwent five cycles of percutaneous ethanol injection therapy in six months, but

**Table 1** Four patients receiving treatments for hepatic metastases

| *Case* | *Treatment* | *Hepatic metastases* | *Histologic radicality* | *Stage (p-TNM)* | *Prognosis* |
|---|---|---|---|---|---|
| 1 | 1. Partial hepatectomy<br>2. Partial hepatectomy | 1. $S_6$<br>2. $S_6$ | non radical | III | alive<br>(5 yr. 11 mon.) |
| 2 | PEIT 5 times<br>Partial hepatectomy | $S_6$<br>multiple | non radical | III | died<br>(2 yr. 6 mon.) |
| 3 | PEIT 2 times<br>CDDP 100mg/Lip 10mL(HA*) | $S_{4,5}$<br>multiple | radical | III | died<br>(6 mon.) |
| 4 | PEIT 2 times | $S_{2,4}$<br>multiple | radical | III | died<br>(11 mon.) |

*Hepatic arterial injection was performed by Seldinger's method.
PEIT = percutaneous ethanol injection therapy; CDDP = cisplatin; Lip = lipiodol; HA = hepatic arterial injection the size of the tumor increased. No evidence of local recurrence was present at that time, so a radical partial resection of the liver and the right lung was performed simultaneously. Pathological findings showed a moderately differentiated tubular adenocarcinoma. A CT scan two months later, however, revealed a coin lesion in the upper lobe of the left lung, so he underwent a radical partial resection of the left lung. Two years and six months after the first operation, he died of multiple metastases in the liver and both lungs.

### Case 3

A 70-year-old man was diagnosed as having cancer of the head of the pancreas, and he underwent the combined therapy. The tumor was a stage III moderately differentiated tubular adenocarcinoma (pT2, pN1, pM0). This operation was classified as a radical operation histologically. A CT scan two months later showed low density areas in liver segments IV and V. Since it was not entirely clear whether these lesions were well localized and therefore resectable, the patient was treated with 2 cycles of percutaneous ethanol injection therapy and an intra hepatic arterial injection of cisplatin (100 mg/10 ml lipiodol) over a two-month period. During this time, the size and number of these tumors increased, and he died six months after the operation.

### Case 4

A 62-year-old man was diagnosed as having cancer of the head of the pancreas, and he underwent the combined therapy. By histopathology, the tumor was a stage III moderately differentiated tubular adenocarcinoma (pT2, pN1, pM0). This operation was classified as a radical operation histologically. A CT scan five months later showed multiple low density areas in segments II and IV of the liver. These tumors were not localized, so a resection was not performed. The patient received 2 cycles of percutaneous ethanol injection therapy. These therapies were not effective, and number and size of the hepatic tumors increased. He died 11 months after the operation.

### DISCUSSION

Despite recent advances in diagnostic and surgical techniques for pancreatic cancer, the long-term survival of patients following resection remains poor. Even following radical resection, a high proportion of patients have local recurrence and hepatic metastases at autopsy. Therefore, for prevention of local recurrence, intraoperative radiotherapy (IORT) was combined with resection at the Kumamoto University School of Medicine since 1976[3]. However, the results when combined with conventional surgery have proved disappointing. Extended radical resection has been combined with extended IORT at our institute since 1984. We have had a dramatic improvement in long-term survival and control of local recurrence compared with other approaches[2]. Although cases 1 and 2 presented here had macroscopically complete tumor clearance, cancer cells were found at the resection margin of the specimen microscopically. Local recurrence, however, was not detected clinically after the combined therapy, indicating the effectiveness of the intraoperative radiation. However, this combined therapy did not prevent hepatic metastases.

Hepatic metastases following a radical operation for pancreatic cancer can fall into one of three patterns: 1) postoperative hepatic metastases, probably due to surgical manipulation, 2) one or more metastatic lesions from local recurrence after resection, or 3) an erroneous preoperative diagnosis. Whatever the cause, a strategy for treating postoperative hepatic metastatic lesions must be devised. In this series, we found one or more hepatic metastases in 8 patients. Multiple hepatic metastases occurred in 6 patients and they died within a year. Therefore, a prudent approach is to observe the patient for several months, perhaps treating with percutaneous ethanol injection therapy, intra-hepatic arterial chemotherapy, or nothing at all. The appearance of new lesions during this time might make the patient inoperable and unlikely to benefit from further surgery. Although percutaneous ethanol injection therapy is an effective treatment for hepatocellular carcinoma[4], it may not be effective for hepatic metastases from pancreatic cancer. One reason may be that it is too solid to inject a sufficient volume of ethanol to induce necrosis so it may be necessary to inject it not only into the lesions, but also into sites around metastases. In cases of multiple hepatic metastases, our results indicate that surgical resection is ineffective. In patients with a solitary tumor, and no evidence of a local recurrence, in good health and able to tolerate the operation, resection of the metastatic lesion may result in long term survival.

Therefore, if local recurrence can be controlled by a radical resection and intraoperative radiation therapy, localized resectable hepatic metastases in the post-operative period should be resected. This will result in some long-term pancreatic cancer survivors. Regarding the implantation and growth of tumor cells released into the portal system, these may be promoted during surgery because of immunosuppression and/or impaired coagulation induced by surgical trauma. Immunochemotherapy may someday be useful for the prevention of hepatic metastases. At present, however, there is no effective treatment for this. We believe that treatments designed to eliminate metastases in the liver during the perioperative period will become an important component of an effective treatment protocol for pancreatic cancer.

In conclusion, if patients with pancreatic cancer have localized' liver metastases after this combined therapy and no local recurrence, their localized liver metastases should be resected to improve their chances for long-term survival.

### REFERENCES

1. Watanapa, P. and Williamson, R. C. N. (1992) Surgical palliation for pancreatic cancer: Developments during the past two decades. *British Journal of Surgery*, **79**, 8–20.
2. Hiraoka, T., Uchino, R., Kanemitsu, K., Toyonaga, M., Saitoh, N., Nakamura, I., Tashiro, S., and Miyauchi, Y. (1990) Combination of intraoperative radiation with resection of cancer of the pancreas. *International Journal of Pancreatology*, 7, 201–207.
3. Hiraoka, T., Watanabe, E., Mochinaga, M., Tashiro, S., Miyauchi, Y., Nakamura, I., and Yokoyama, I. (1984) Intraoperative irradiation combined with radical resection for cancer of the head of the pancreas. *World Journal of Surgery*, **8**, 766–771.
4. Seki, T., Nonaka, T., Kubota, Y., Mizuno, T., and Sameshima, Y. (1989) Ultrasonically guided percutaneous ethanol injection therapy for hepatocellular carcinoma. *The American Journal of Gastroenterology*, **84**, 1400–1407.

